# Functional Characterization of a Dual Enhancer/Promoter Regulatory Element Leading Human *CD69* Expression

**DOI:** 10.3389/fgene.2020.552949

**Published:** 2020-10-27

**Authors:** Jennifer Redondo-Antón, MG Fontela, Laura Notario, Raúl Torres-Ruiz, Sandra Rodríguez-Perales, Elena Lorente, Pilar Lauzurica

**Affiliations:** ^1^Immune Gene Regulation and Antigen Presentation Group, National Center for Microbiology, Institute of Health Carlos III (ISCIII), Madrid, Spain; ^2^Molecular Cytogenetics and Genome Editing Unit, Spanish National Cancer Research Centre (CNIO), Madrid, Spain

**Keywords:** CD69, immune regulation, enhancer, promoter, enhancer-derived RNA (eRNA), transcriptional regulation

## Abstract

The *CD69* gene encodes a C-type lectin glycoprotein with immune regulatory properties which is expressed on the cell surfaces of all activated hematopoietic cells. *CD69* activation kinetics differ by developmental stage, cell linage and activating conditions, and these differences have been attributed to the participation of complex gene regulatory networks. An evolutionarily conserved regulatory element, CNS2, located 4kb upstream of the *CD69* gene transcriptional start site, has been proposed as the major candidate governing the gene transcriptional activation program. To investigate the function of human CNS2, we studied the effect of its endogenous elimination via CRISPR-Cas9 on CD69 protein and mRNA expression levels in various immune cell lines. Even when the entire promoter region was maintained, CNS2-/- cells did not express CD69, thus indicating that CNS2 has promoter-like characteristics. However, like enhancers, inverted CNS2 sustained transcription, although at a diminished levels, thereby suggesting that it has dual promoter and enhancer functions. Episomal luciferase assays further suggested that both functions are combined within the CNS2 regulatory element. In addition, CNS2 directs its own bidirectional transcription into two different enhancer-derived RNAs molecules (eRNAs) which are transcribed from two independent transcriptional start sites in opposite directions. This eRNA transcription is dependent on only the enhancer sequence itself, because in the absence of the *CD69* promoter, sufficient RNA polymerase II levels are maintained at CNS2 to drive eRNA expression. Here, we describe a regulatory element with overlapping promoter and enhancer functions, which is essential for *CD69* gene transcriptional regulation.

## Introduction

Gene expression is coordinated by an interplay among different regulatory regions, which work together to determine precisely when, where, and how they are transcribed. The orchestration of gene transcriptional programs relies on two main players: promoters and distal regulatory elements. Whereas promoters are located immediately upstream of gene transcriptional start sites (TSS), distal regulatory elements act over long genomic distances in an orientation-independent manner. Promoters are thought to be sufficient to recruited RNA polymerase II (POLR2A) and establish the start site and directionality of transcription. Basal expression from promoters is modulated by a combination of signals from distal regulatory elements, which determine the final transcriptional output.

Traditionally, enhancers and promoters have been considered to be two different regulatory elements on the basis of chromatin characteristics, such as DNase hypersensitivity and chemical modification of histone tail residues. Promoters are characterized by open chromatin regions bearing H3K4me3 whereas H3K27ac, H3K4me1 and P300 are often associated with enhancers (Heintzman et al., 2007; Visel et al., 2009). High-throughput screens have revealed many similarities in structure and functionality between both regulatory features. Indeed, well known enhancer elements can act as promoters (De Santa et al., 2010; van Arensbergen et al., 2017) and many gene promoters display distal effects on the regulation of different genes (Dao et al., 2017; Diao et al., 2017), thus behaving as enhancers. Consequently, current trends support a unique regulatory feature with varying degrees of overlap of promoter and enhancer potential (Andersson and Sandelin, 2020).

Distal regulatory elements control promoter activity, but the mechanisms underlying this functional interaction remain unclear. Described enhancer roles include pre-initiation complex activation, promoter chromatin remodeling, and the transition of paused to active POLR2A or its direct recruitment (Beagrie and Pombo, 2016). Recently, RNA molecules transcribed from enhancers, termed enhancer-derived RNAs (eRNAs), have been found to play an active role in the development of the enhancer function (Lam et al., 2014). Enhancers are frequently bidirectionally transcribed into non-coding RNAs with short half-lives, which are generally not spliced or polyadenylated, and are typically short (1–2 Kb) and retained in nuclear fractions (Lewis et al., 2019). These molecules, in addition to be markers of active enhancers (Andersson et al., 2014a), act in *cis* or *trans* (interchromosomally) (Tsai et al., 2018) in regulating the expression of target genes. Depletion of eRNAs often results in downregulation of nearby gene expression and, although different mechanisms of action have been proposed (Lewis et al., 2019), several studies support that this effect is due to the inhibition of chromatin looping, which brings promoters and distal regulatory elements into contact, as revealed by proximity ligation techniques (3C, 4C and HiC) (Hsieh et al., 2014; Pnueli et al., 2015).

CD69 is an immune regulatory protein that is present on the surfaces of all hematopoietic cells after activation. This membrane molecule modulates immune responses through controlling cytokine and chemokine production, and regulates lymphocyte egress from lymphoid organs via S1PR1 posttranscriptional downregulation (Bankovich et al., 2010; Notario et al., 2018). *In vivo* studies in CD69 KO mouse models have demonstrated an important role of CD69 in the pathogenesis of different inflammatory and autoimmune diseases (Radulovic and Niess, 2015; Cibrian and Sanchez-Madrid, 2017), and a critical function in the anti-infectious (Vega-Ramos et al., 2010; Notario et al., 2016, 2019), anti-tumoral (Esplugues et al., 2003, 2005; Mita et al., 2018) and airway inflammatory responses (Hayashizaki et al., 2016; Kimura et al., 2017).

The *CD69* gene is located on human chromosome 12 as part of the NK complex, together with many other genes encoding lectins with varying cell-type specific functions in the immune system. The *CD69* proximal promoter contains the canonical TATA box and binding sites for early response inducible transcription factors, such as NFκB and AP1 (Santis et al., 1994; Lopez-Cabrera et al., 1995). Analysis of the *CD69* 5′ region has revealed conservation at four genomic domains denoted as CNS1-4 (Conserved Non-coding Sequence) relative to the promoter. These four elements display DNase hypersensitivity and bear regulatory cell-type specific histone marks that undergo changes during development and activation (Vazquez et al., 2009). Transient transfection and transgenesis assays have suggested a major role of the CNS2 element, located 4kb upstream of the human CD69 TSS, which is occupied by dense clusters of transcription factors (Laguna et al., 2015; Fontela et al., 2019) and has strong *in vivo* regulatory potential (Mumbach et al., 2017; Simeonov et al., 2017).

In this study we further characterize the human CNS2 regulatory element, which displays both promoter and enhancer functions, and can direct its own transcription independently of the activity of its target gene. By using the CRISPR-Cas9 technology to delete or invert the endogenous CNS2 element, we identified its promoter function and its ability to potentiate transcription, which is partially retained when it is present in an inverted orientation. As a promoter, CNS2 recruits POLR2A and directs its own transcription into two different eRNAs, independently of the presence of the *CD69* promoter or its activity.

## Materials and Methods

### Cell Culture and Flow Cytometry

Jurkat T cells and C1R B cells were cultured in RPMI medium supplemented with 10% fetal calf serum, 1% L-glutamine and 100 U/ml of penicillin-streptomycin (Gibco). Cells were cultured under standard conditions (37°C, 5% CO_2_).

To determine the CD69 surface expression levels in different assayed conditions, we cultured 10^6^ cells with or without 10 ng/ml PMA and 500 ng/ml ionomycin for 24 h. After incubation, cells were stained with PE-conjugated mouse anti-human CD69 antibody (clone FN50, Biolegend, 310906) for 20 min at 4^*o*^C and analyzed with a FACSCanto flow cytometer (Becton, Dickinson, Franklin Lakes, NJ, United States) and FACSDiva software (Becton, Dickinson). Data were analyzed in FlowJo (TreeStart Inc., Ashland, OR, United States).

### Luciferase Assays

DNA was isolated from Jurkat T cells with 10% SDS-containing buffer followed by ethanol purification. The *CD69* promoter and CNS2 region were PCR amplified with NZYTaq II 2x Colorless Master Mix (NZY; MB357). The primer sequences used are listed in Table 1. The *CD69* promoter or CNS2 amplification products were cloned into the pGL3b plasmid (Promega; E1751) upstream of the luciferase ORF with Gibson Assembly 2x Master Mix (NEB; E2611), according to the manufacturer’s instructions. *Nco*I-digested pGL3b plasmid and PCR-amplified fragments containing vector-overlapping ends, were mixed 1:1 with Gibson Assembly Master Mix and incubated for 10 min at 50°C. The CNS2 region was cloned into the pGL3b-*CD69* promoter-containing vector via the *Hin*dIII restriction site. Cloning products were transformed into DH5α competent *E. coli* cells (NZY; MB004). Vector integrity and fragment directionality were confirmed by Sanger sequencing. The plasmids with the correct sequence were purified with Qiagen Mini Kit and used for transfection.

**TABLE 1:** PCR primers used for CD69 promoter or CNS2 insertion into pGL3b plasmid using Gibon Assembly Master Mix.

Name	Sequence (5′-3′)
pGL3b_promoter_fw	ggtactgttggtaaagccaccatggTCCAAAAACCAATT CGTAGCTTTC
pGL3b_promoter_rw	tttatgtttttggcgtcttccatgGTGAGGCTCTGA GGCATC
pGL3b_promoter_inv_fw	ggtactgttggtaaagccaccatggGTGAGGCT CTGAGGCATC
pGL3b_promoter_inv_rw	tttatgtttttggcgtcttccatggTCCAAAAACCAATTC GTAGCTTTC
pGL3b_CNS2dir_ fw	ggtactgttggtaaagccaccatggTATGGTACATTTGCTAT TTTCAC
pGL3b_CNS2dir_ rw	tttatgtttttggcgtcttccatggACCTCTTTATAAAACA CAGATG
pGL3b_CNS2inv_ fw	ggtactgttggtaaagccaccatggACCTCTTTATAAAACA CAGATG
pGL3b_CNS2inv_ rw	tttatgtttttggcgtcttccatggTATGGTACATTTGCT ATTTTCAC
pGL3B-prom-CNS2dir_fw	cgagatctgcgatctaagtaTATGGTACATTTGCTAT TTTCAC
pGL3B-prom-CNS2dir_rw	ccaacagtaccggaatgccaACCTCTTTATAAAACAC AGATG
pGL3B-prom-CNS2inv_fw	cgagatctgcgatctaagtaACCTCTTTATAAAACA CAGATG
pGL3B-prom-CNS2inv_rw	ccaacagtaccggaatgccaTATGGTACATTTGCTA TTTTCAC

Jurkat T cells were transfected with 100 ng of each construct and 2 ng of the Renilla plasmid (pRL-TK, Promega), which was used as an internal control for transfection efficiency. Electroporation was performed with the Neon transfection System (Thermo Fisher) according to the manufacturer’s instructions. One day after electroporation, cells were stimulated with 10 ng/ml phorbol 12-myristate 13-acetate (PMA) and 500 ng/ml ionomycin for 24 h. The cells were then lysed and luciferase activity was measured with a Dual luciferase kit (Promega E1941, Madison, WI, United States) and an Orion II microplate luminometer (Berthold 11300010, Bad Wildbad, Germany). The activity of each vector was expressed as the ratio between firefly and Renilla luciferase luminescence. Data are presented as mean ± SEM of three different experiments, each with two technical duplicates.

### CRISPR-Cas9 Deletions

**FIGURE 1:**
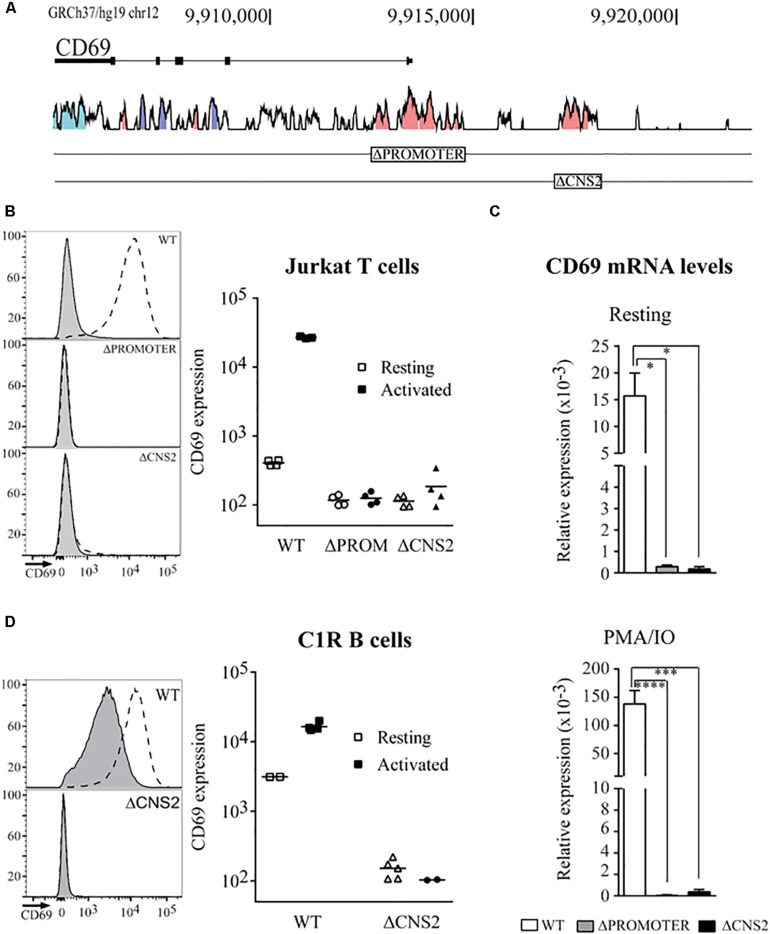
Deletion of the CNS2 regulatory element impairs CD69 mRNA and protein expression. **(A)** Vista browser tracks (http://pipeline.lbl.gov/cgi-bin/gateway2) showing conservation between the human and mouse *CD69* gene locus (GRCh37/hg19 assembly, chr12:9,905,077-9,920,582). Blue = untraslated regions, Purple = exons, Pink = conserved non-coding regions. Boxes indicate the deleted regions. **(B,D)** Flow cytometry analysis of CD69 surface expression in Jurkat T Cells **(B)** or C1R B cells **(D)** Left. Representative histograms of the expression profiles for Promoter or CNS2 deletion in cells stimulated (dotted line) or not stimulated (gray line) with PMA/IO for 24 h. Right. Mean fluorescence intensity (MFI) of different wild-type (squares), ΔPromoter (circles) or ΔCNS2 (triangles) clones, either unstimulated (empty) or stimulated (filled). **(C)**. *CD69* mRNA levels in basal conditions and after 3 h PMA/IO stimulation in WT, ΔPromoter, and ΔCNS2 conditions. Data represent the mean ± SEM of three experiments using three different clones. ns: non-significant, *p < 0.05, **p < 0.01, ***p < 0.001, ****p < 0.0001.

The CRISPR-Cas9 system was used to eliminate the promoter and the CNS2 sequences by deletion. Two simultaneous double strand breaks resulted in a loss of the intervening DNA sequence without large insertions/deletions via non-homologous end-joining repair (Zheng et al., 2014). Sequences upstream and downstream of the promoter and the CNS2 regions were analyzed for CRISPR-Cas9 targets with the CRISPR Design tool available on-line at https://benchling.com/. The selected sequences and their genomic positions are listed in Table 2 and shown in Figure 1A.

**TABLE 2:** Sequence and genomic position of each sgRNA used for promoter or CNS2 CRISPR-Cas9 deletion.

Name	Sequence (5′-3′)	Genomic position (GRCh37/hg19)
Prom_5′_gRNA1	GGATGCTGTCATG AGAACAC	Chr12:9912818-9912838
Prom_3′_gRNA2	CATAGCAGCTAGAA CCATTG	Chr12:9914667-9914687
CNS2_5′_gRNA1	CGGTACTAATCAATA CTTGT	Chr12:9916938-9916958
CNS2_3′_gRNA2	TGTGTGCACCTAACA TACCT	Chr12:9918359-9918379

Specific crRNAs, tracrRNA, and Cas9-3NLS were ordered from Integrated DNA Technologies. To produce guide-RNAs, we mixed crRNA and tracrRNA, heated them at 80°C for 10 min and cooled them to room temperature. To generate CRISPR-Cas9 ribonucleoproteins, we incubated sgRNAs with Cas9 protein at 1:1 ratio for 20 min. For each transfection, approximately 2 × 10^5^ cells were co-transfected with Prom 3′sgRNA and Prom 5′sgRNA or CNS2 3′sgRNA and CNS2 5′sgRNA with the Neon Transfection System (Torres-Ruiz et al., 2017). The electroporation parameters used for each cell type are described in Table 3. Single cell clones were isolated and screened via PCR using primer pairs mapping inside and outside the deleted region. Several homozygous, heterozygous, inverted and wild-type clones were selected for further analysis.

**TABLE 3:** Electroporation conditions used for CRISPR-Cas9 system transfection.

Cell type	Pulse voltage (V)	Pulse width (ms)	Pulse number
Jurkat (JJ)	1325	10	3
C1R	1450	10	3
Primay T cells	1600	10	3

### RNA Extraction and RT-qPCR

RNA from CRISPR-Cas9-deleted cell lines was isolated with NZY total RNA isolation kit (MB13402). cDNA synthesis was performed with a combination of random hexamers and oligodT primers with an NZY First Strand cDNA Synthesis Kit (MB125). Gene expression was evaluated by probe-based qPCR analysis [NZY qPCR Probe Master Mix (2x), MB227] on QuantStudio3 (Thermo Fisher) by using a predesigned qPCR assay from Integrated DNA Technologies (IDT) (Assay ID: Hs.PT.58.20340459). Human β2microglobulin (assay ID: Hs.PT.58v.18759587) mRNA expression was used for normalization. Relative gene expression was calculated with the ΔCt method.

### eRNA Analysis by qPCR

Total RNA was obtained with Trizol (Thermo Fisher, 15596026). After extraction, the RNA was treated with RQ1 RNase-free DNAse (Promega, M6101) for 1 h at 37°C with 1 μl of DNase per μg RNA in a total volume of 50 μl. The RNA was then re-purified with phenol/chloroform and precipitated with 70% ethanol. A total of 3–5 μg of RNA was reverse-transcribed in a total volume of 20 μl with random primers and SuperScript II reverse transcriptase (Thermo Fisher) for 1 h. Because eRNAs do not undergo splicing, an identical reaction of each sample, with the same quantity of RNA, but without RT enzyme (-RT control), was used to evaluate DNA contamination. The cDNA was diluted to 100 ng/μl on the basis of the initial RNA input. A volume of 1 μl of diluted cDNA was used in a 10 μl qPCR reaction with NZY qPCR Green Master Mix 2x (MB221). Quantification was performed on QuantStudio3 (Thermo Fisher) with the primers listed in Table 4. Human β2microglobulin was used for normalization. The average of two technical duplicates was used for all quantifications. Relative gene expression was calculated using the ΔCt method.

**TABLE 4:** Primers sequences used for sense and antisense eRNA quantification.

Name	Sequence (5′-3′)
β2microglobulin_fw	GGACTGGTCTTTCTATCTCTTGT
β2microglobulin_rw	ACCTCCATGATGCTGCTTAC
antisenseRNA_fw	TCAAGAAAACACTGCAATCAAAA
antisenseRNA_rw	TGTCAATGTGGCAAGTTGTG
senseRNA_fw	TGCAAGAGCCTGAACTTGTTG
senseRNA_rw	GCATGTGTCAGCTAGGACAGT

### Chromatin Immunoprecipitation (Chip)

ChIP experiments were performed with a Chromatrap Pro A Chip-seq Sonication 24 column kit (Chromatrap; 500189), according to the manufacturer’s recommendations. Briefly, 1 × 10^7^ cells, treated for 30 min with PMA + IO, washed and fixed with 37% paraformaldehyde for 10 min. The cross-linking reaction was quenched with 1,375 M glycine before cell lysis. For chromatin sonication, 20 pulses of 30 s were applied with Bioruptor Next Generation instrument (Diagenode). Sheared chromatin was incubated with 10 μg of the following antibodies: anti-RNA polymerase II CTD repeat YSPTSPS (phospho S2) (ABCAM, ab5095); antiH3K4me3 (ABCAM, ab8580) and anti-IgG (Cell signaling, 2729); at 4°C overnight. Chromatrap Spin Columns were used for immunoprecipitation. DNA from the eluted immunocomplexes was purified with a QIAquick PCR Purification Kit (Qiagen) columns. Real-time PCR analysis was performed with QuantStudio3 (Thermo Fisher) and NZY qPCR Green Master Mix, ROX plus (NZY MB222). The Ct values of IgG controls were used for normalization. Primer sequences (Table 5) were designed with Blast Primer and validated via qPCR with DNA from ΔPROMOTER and ΔCNS2 DNA to avoid non-specific amplification. Amplicons verified with melting curve analysis and gel electrophoresis.

**TABLE 5:** Primers sequences used for Chip analysis.

Name	Sequence (5′-3′)
CNS2Chip_fw	TGTAGAATCCAGGGTGAGACG
CNS2Chip_rw	AGAGATGAGCAGTTTGTCTCCG
CD69promChip_fw	GCTGGAGCTCTTGTTGAGTCT
CD69promChip_rw	CAAGCAAGTAGGCGGCAAGA

### RACE Reactions

Characterization of CNS2 non-coding transcripts was performed with a GeneRacer RACE kit from Thermo Fisher Scientific according to the manufacturer’s instructions. In brief, 5 μg of total RNA from 30 min stimulated Jurkat T cells was treated with tobacco acid phosphatase to remove 5′cap and expose 5′ phosphates, thus enabling the ligation of the GeneRacer RNA Oligo with a known specific sequence. cDNA was generated by reverse transcription with SuperScript II RT and either random hexamers or GeneRacer Oligo dT primer. A 1 μl volume from a 1/5 dilution of the RACE product was used in PCRs with GeneRacer 5′ Primer annealing to 5′ GeneRacer RNA Oligo and different gene specific 3′ primers in a 20 μl total reaction. To avoid non-specific bands, we re-amplified 1μl of PCR product with GeneRacer 5′ Nested Primer and a second inner specific primer. Only cDNA containing the GeneRacer RNA oligo was amplified. A similar procedure was followed for 3′ end characterization, with GeneRacer 3′ primer and GeneRacer 3′ Nested primer. Amplification bands were analyzed by Sanger sequencing. Specific primer sequences used for sense and antisense-RNA characterization are listed in Table 6.

**TABLE 6:** Primers sequences used for the analysis of sense and antisense eRNA 3′ end.

Name	Sequence (5′-3′)
eRNA_antisense_F_rw	TGGTACATTTGCTATTTTCACACCA
eRNA_antisense_I_rw	TGTCAATGTGGCAAGTTGTG
eRNA_antisense_J_rw	TTCGACCCTTCTCCCCAATC
eRNA_antisense_K_rw	GGATGAAAGCTTAAAGGGGC
eRNA_antisense_L_rw	AGCCATTAGGGAAATGCAAA
eRNA_antisense_M_rw	TTAGTGTGGGGGCAGAATCTT
eRNA_antisense_N_rw	TTTGATTGGCTTTGCCTCTT
eRNA_antisense_O_rw	TGCATCCACTACACTGTTCAGA
eRNA_sense_1_fw	CTGTCCTAGCTGACACATGC
eRNA_sense_2_fw	TGCAAGAGCCTGAACTTGTTG
eRNA_sense_3_fw	ATCAGTACTTGGGTGCGTGG

### Circular RNA

To determine the 3′ end of the different eRNA species transcribed from the CNS2 region, we used a previously described RNA circularization method (Couttet et al., 1997; Kim et al., 2010). Briefly, 5μg of DNase treated RNA were incubated with 0.5 units of tobacco acid pyrophosphatase (Thermo Fisher) at 37°C for 1 h to expose the phosphates at the 5′ ends of all RNAs. Decapped RNA was purified and circularized with 5 units of T4 RNA ligase, which catalyzed phosphodiester bond formation between the generated 5′ phosphate and the 3′ hydroxyl group. cDNA synthesis from circularized RNA was performed with SuperScript III and random hexamers with the following program: 25°C for 5 min, 50°C for 1 h and 70°C for 15 min. After reverse transcription, 1μl of cDNA was used to amplify 5′-3′ junctions with primer pairs specifically design to that end (Supplementary Figure 1A). For sequencing, PCR products were cloned with TOPO-TA cloning kit (Thermo Fisher, K4575J10) according to manufacturer’s instructions.

### Statistical Analysis

All data was represented and analyzed with GraphPad Prism 7 software. Significant differences between the different assayed conditions were tested with an unpaired two-tailed t-test. Differences with *p* < 0.05 were considered as statistically significant: ^∗^*p* < 0.05, ^∗∗^*p* < 0.01, ^∗∗∗^*p* < 0.001, ^****^p < 0.0001. Non-significant differences are indicated as ns.

## Results

### The CNS2 Regulatory Element Is Necessary but Not Sufficient for CD69 Expression

On the bases of transient transfection experiments and transgenesis assays, we previously defined four non-coding regions upstream of the CD69 promoter as key regulatory elements required for proper gene transcriptional regulation during T cell development and activation (Vazquez et al., 2009). ENCODE data, together with our recent work (Fontela et al., 2019) and that of others (Mumbach et al., 2017; Simeonov et al., 2017) suggest that CNS2, located 4 kb upstream of human CD69 TSS, as the major candidate governing the gene transcriptional activation program.

To further investigate CNS2 function, we applied CRISPR-Cas9 technology to delete 1432 bp of DNA containing the entire conserved CNS2 region (chr12:9916938-9918379; human Feb 2019). We obtained several cell lines with homozygous deletion of the CNS2 element (CNS2-/-) and also produced cell lines with a promoter deletion (chr12:9912818-9914687; PROM-/-) as a negative control for CD69 expression (Figure 1A).

To assess the effects of CNS2 deletion on CD69 expression, we measured surface protein levels in basal conditions and at their peak after activation with PMA/IO for 24 h. For Jurkat T cells (Figure 1B), very low levels of CD69 protein were detected in unstimulated conditions (MFI = 408), whereas PMA/IO treatment led to a 60-fold increase over basal conditions (MFI = 26900). As predicted, promoter-deleted clones displayed lower basal CD69 levels (MFI = 117) and an inability to respond to stimulation (MFI = 125). Unexpectedly, the effects of the lack of the CNS2 regulatory region resembled those of promoter deficiency, in which CD69 expression was abolished both in the basal state (MFI = 114) and after PMA/IO stimulation (MFI = 185). In agreement with the protein data, only residual levels of CD69 mRNA were detected in both cases at steady state and after 3 h stimulation (Figure 1C). When we analyzed the effect of CNS2 deletion in the C1R B cell line (Figure 1D), which constitutively expresses CD69, we observed not only the loss of response to stimulation but also an inability to sustain basal expression.

Together, these results indicate that CNS2 acts as part of the promoter and theirs cooperative function is necessary for transcription, since none of them can act on their own to induce CD69 expression.

### The CNS2 Element Has Overlapping Promoter and Enhancer Functions

**FIGURE 2:**
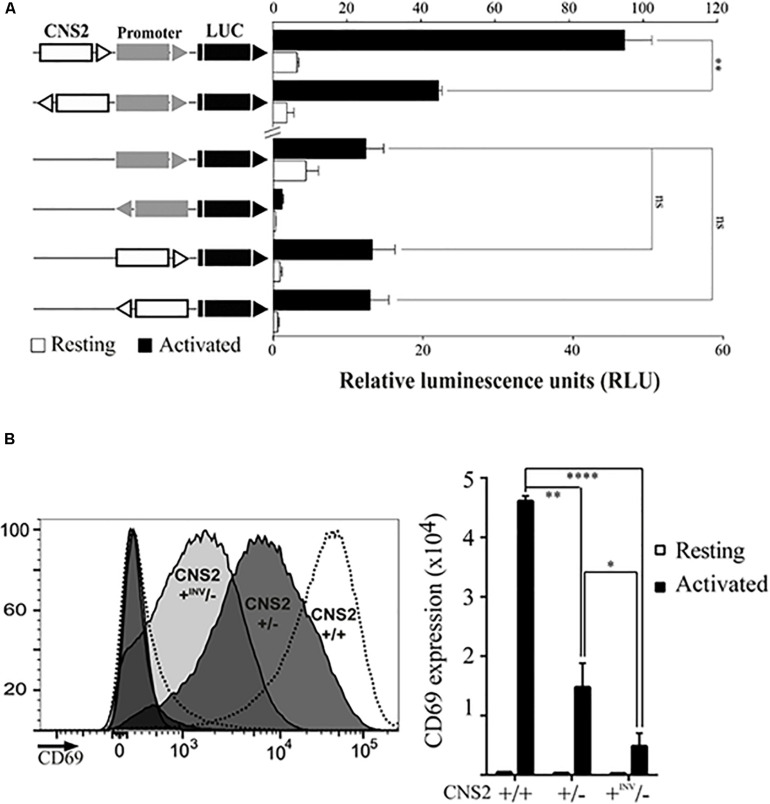
CNS2 activity is partially orientation-independent. **(A)** Jurkat cells were transfected with luciferase constructs bearing the CD69 promoter and/or CNS2 regulatory elements in the indicated positions and orientations with respect to the luciferase ORF. Bars and error bars denote the mean ± standard error of the mean (SEM), respectively, of relative luminescence units from three individual experiments, being the value of each experiment the mean of two technical duplicates. **(B)** Left. Histograms of CD69 surface expression 24 h post-activation in representative CNS2 ± (dark gray) and CNS2 + ^inv^/- (light gray) clones. Histograms of non-activated and activated wild type controls are represented as dashed lines. Right. MFI of CD69 surface expression in two independent CNS2 ± clones and four CNS2^inv^/- clones in basal conditions (white) or 24 h post-activation (black). Mean ± SEM of CD69 MFI of the various assayed clones are shown. ns: non-significant, *p < 0.05, **p < 0.01, ***p < 0.001, ****p < 0.0001.

To further investigate the role of CNS2 in CD69 transcriptional activation we assayed its ability to work independently of its orientation, as traditionally described for distal regulatory elements. Thus, we examined whether an inverted CNS2 might enhance CD69 promoter activity in a luciferase reporter assay (Figure 2A). The CD69 promoter alone directed low levels of luciferase expression and conferred a 3-fold increase in expression under stimulation conditions. Addition of CNS2 induced promoter activity increases of 2-fold and 10-fold in the basal and stimulated states, respectively. The inverted CNS2 retained basal activity and upregulated CD69 after activation up to half the expression level reached with its regular orientation. We also tested the ability of CNS2 to direct promoter-less luciferase expression in both directions. The CNS2 regulatory element itself directed luciferase expression at similar levels to those of the promoter alone. In contrast to the results for the CD69 promoter, the inverted CNS2 element alone completely retained its activity.

We also measured the effect of CNS2 inversion in its original genomic context, by analyzing CD69 expression in different CNS2-/ + edited clone, which were found to contain the remaining CNS2 copy in an inverted position (Figure 2B). We detected this rearrangement with a forward primer annealing outside the deleted region and a second forward primer annealing inside it. Sanger sequencing confirmed the CNS2 inversion between both CRISPR-Cas9 targets. Approximately, 30% of activation-induced CD69 expression was retained when only one CNS2 copy drove transcription (CNS2 ±). When in heterozygous conditions, the CNS2 element was in an inverted position (+ INV/-), the expression was 70% lower than that in wild-type ± counterparts. Consequently, even when CNS2′s orientation-independent activity was maintained, its ability to activate transcription in these circumstances was diminished.

Therefore, a combination of experiments in genomic and episomal contexts supports the convergence of traditional promoter and enhancer properties in the CNS2 regulatory element.

### Bidirectional Transcription of CNS2 Regulatory Element

High-throughput analysis of gene regulatory regions have revealed that distal elements, preferentially those marked with H3K4me1 and H3K27ac, are commonly transcribed bidirectionally into a newly described class of RNA molecules.

**FIGURE 3:**
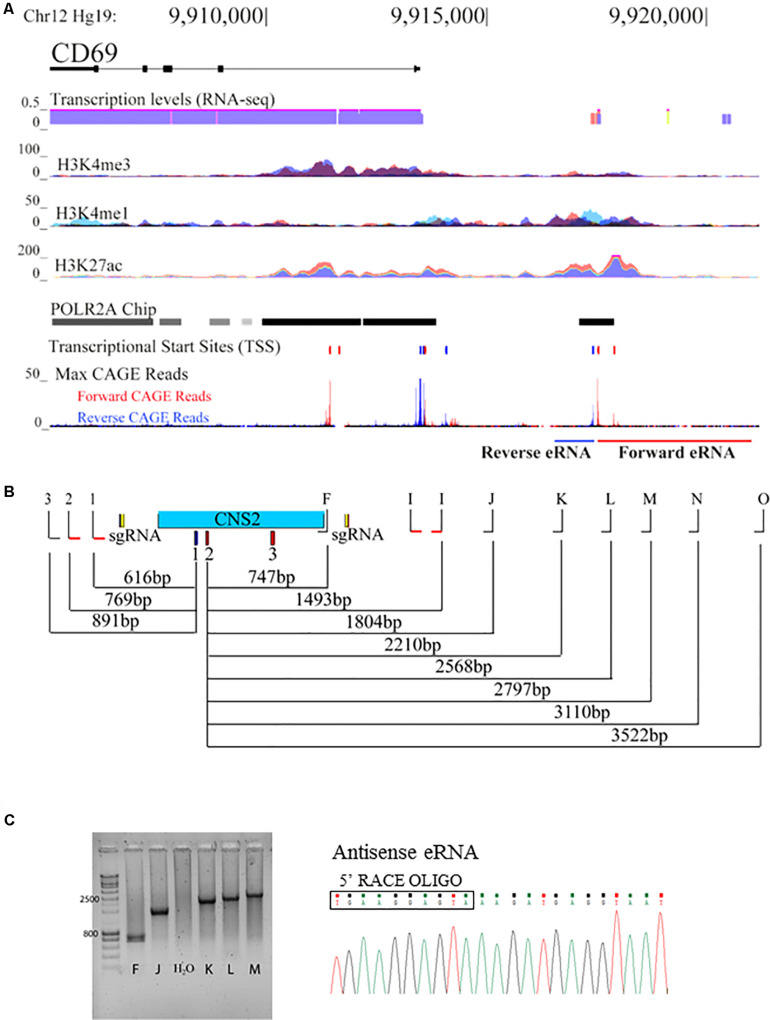
CNS2 displays promoter activity in its genomic context. **(A)** ENCODE data (GRCh37/hg19 assembly, chr12:9,905,077-9,920,582) for transcription levels (from RNA-seq data), and binding of H3K4me3, H3K4me1, H3K27ac and POLR2A at the CD69 locus. The locations of the different TSS and mapped CAGE-reads are also represented. **(B)** Locations of the different primers used to assay sense and antisense eRNA lengths with respect to the different TSS within the CNS2 region. **(C)** Agarose gel of the PCR products obtained from the amplification of RACE-treated RNA from 30 min PMA/IO activated Jurkat T cells with 5′RACE oligo and primers annealing at different distances along CNS2 for antisense-eRNA. Sequence from one of the PCR products depicts the beginning of the antisense-eRNA molecule, defining its TSS.

In agreement with a putative promoter function of CNS2, publicly available data show overlapping RNA-seq peaks, H3K4me1 and H3K27ac marks and POLR2A binding within this region, which may indicate transcriptional activity (Figure 3A). In addition, Cap Analysis of Gene Expression (CAGE) experiments revealed three potential TSS within the CNS2 sequence; the first, henceforth referred to as sense- TSS (GRCh37/hg19 chr12:9917412-9917425), initiates negative strand transcription toward the CD69 gene coding sequence; the others (GRCh37/hg19 chr12:9917527-9917543 and chr12:9917895-9917913), antisense-TSSs, transcribe the positive strand in the opposite direction (Figure 3A; Andersson et al., 2014a).

Together, these data suggest that the CNS2 element might be transcribed into at least three eRNAs molecules. To explore this possibility we performed 5′ and 3′ Rapid Amplification of cDNA Ends (RACE) reactions on total RNA from 30 min-stimulated Jurkat T cells. 3′ RACE reactions did not reveal transcript 3′ ends, thus suggesting that none of the potential transcripts were polyadenylated, in agreement with evidence indicating a lack of polyadenylation in this type of RNA molecules (Kim et al., 2010). However, 5′ RACE analysis demonstrated the production of two different RNAs from two TSS, separated by 61 bp, in opposite directions. The position of the sense TSS was identical to that predicted, whereas the antisense TSS was shifted 45 bp upstream of its reported position. Then, to assess its complete length, we used a primer extension PCR assay combining 5′ RACE oligo with specific primers at different positions with respect to the TSS (Figure 3B). We stablished the approximate length of each non-coding transcript determining whether a PCR product could be obtained with a primer at a certain position (forward eRNA analysis in Figure 3C). In that way, we observed that the reverse eRNA was between 886–1089 bp long; whereas the forward eRNA was 3522–3762 bp in length. eRNA sequencing confirmed that none of the transcripts underwent splicing. We did not detect activity from the second forward TSS. With a circular RNA method, we detected two sense-RNA species of 896 and 739 bp arising from the same TSS (Supplementary Figure 1A).

**FIGURE 4:**
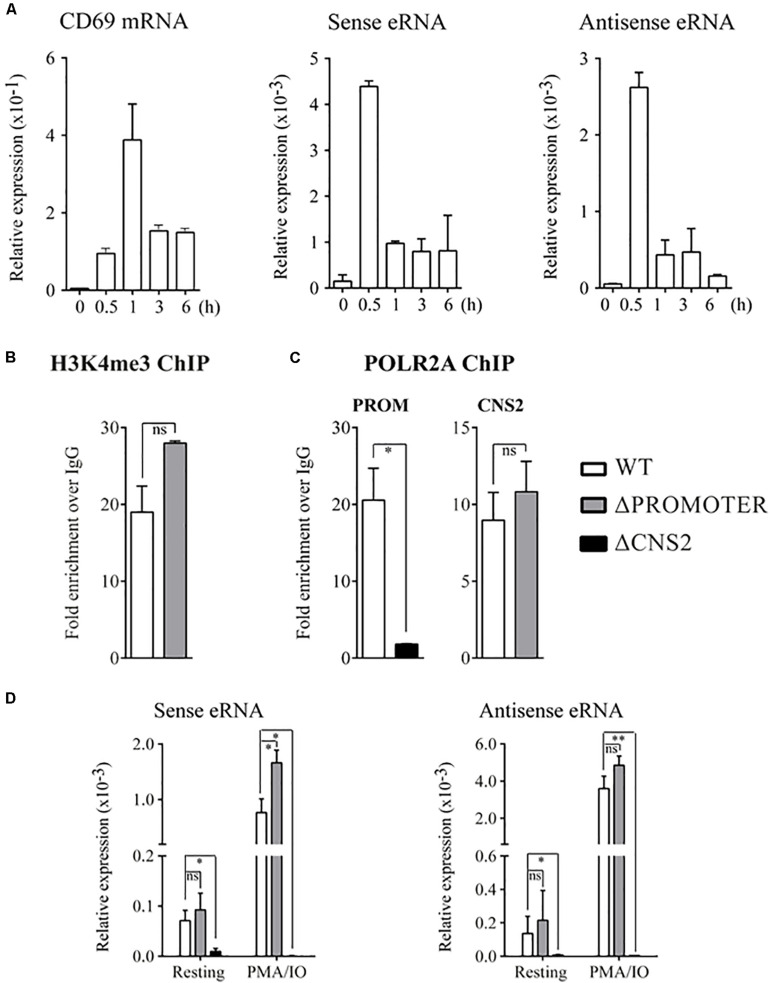
CNS2 eRNA production is independent of the CD69 promoter. **(A)** *CD69* mRNA, sense and anti-sense eRNA kinetics measured in wild type Jurkat T cells after activation were assayed by quantitative PCR. Mean ± SEM of the data of two different experiments is shown. **(B)** H3K4me3 at CNS2 region in WT and ΔPROMOTER Jurkat T cells after 30 min stimulation with PMA/IO. **C** POLR2A binding at the *CD69* promoter or CNS2 element after 30 min-stimulation assayed by ChIP in Jurkat WT, ΔPROMOTER and ΔCNS2 cells. **(B,C)** Each analysis was performed with a mix of three different clones for ΔPROMOTER and ΔCNS2 conditions. Error bars denote the mean ± SEM of two different technical duplicates. **(D)** Quantification of sense and antisense eRNA levels in the basal state and after 30 min-stimulation in WT, ΔPROMOTER, and ΔCNS2 cells. The results of three experiments using different clones are presented as mean ± SEM.ns: non-significant, ^∗^*p* < 0.05, ^∗∗^*p* < 0.01.

Quantification and kinetics analysis of CNS2 transcription was assayed with RT-qPCR. DNase-treated RNA was reverse-transcribed with random primers and quantification was performed with the primer pairs highlighted in red at Figure 3B. eRNA expression was upregulated 50-fold within the first 30 min of PMA/IO exposure, then gradually decreased in a time-dependent manner. The expression kinetics was similar between the sense and antisense transcripts but preceded the induction of CD69 mRNA (Figure 4A), a finding that appears to reflect independent transcription between the nearest coding RNA and both eRNA species.

### Autonomous Promoter Activity of the CNS2 Regulatory Element

Because occupancy by POLR2A and H3K4me3 has been described as a feature of regulatory elements with promoter ability, we analyzed these parameters at the CNS2 region, as well as how they affected by promoter deletion.

Chip experiments of POLR2A binding kinetic at CD69 promoter and CNS2 regulatory element showed a low occupancy at both regions before 30 min of stimulation (Supplementary Figure 1B). Chip experiments of 30 min stimulated WT, PROM-/- and CNS2-/- cell lines are shown in Figures 4B,C. CNS2 occupancy by H3K4me3 was not strikingly affected by promoter deletion (Figure 4B). However, although the absence of CNS2 reduced 9-fold POLR2A binding at the CD69 promoter, lack of CD69 promoter did not affected the ability of CNS2 region to recruit POLR2A (Figure 4C). These results indicate a synergistic action of both elements in the recruitment of appropriate POLR2A levels at CD69 promoter, in a manner that may be dependent on the physical interaction between them.

Next, to explore whether the absence of the promoter affected eRNA transcription at CNS2 region, we measured the forward and reverse eRNA levels in the basal state and after a 30 min induction with PMA/IO in WT, PROM-/- and CNS2-/- cell lines. The detection was performed with qPCR using primer pairs located outside the deleted CNS2 region (Figure 3B, red lines). As shown in Figure 4D, the absence of CD69 promoter did not impair CNS2 bidirectional transcription. The lack of amplification among CNS2-/- samples corroborates eRNA beginning within CNS2 element and confirmed the absence of DNA contamination.

Therefore, these results confirmed that CNS2 regulatory element constitutes an independent transcriptional unit that does not require the assistance of the promoter to produce its own transcripts.

## Discussion

We previously reported a DNase hypersensitive site bearing specific regulatory epigenetic marks and dense clusters of transcription factor binding sites which acts as a potent regulatory element in the context of episomal and transgenesis assays. In this study we targeted the endogenous CNS2 element by CRISPR-Cas9 deletion in various human cell lines and primary T cells to evaluate its function in a native chromatin context. Like promoters, the CNS2 regulatory region is necessary for transcription initiation and promotion, but it displays orientation-independent activity in driving and potentiating transcriptional output, functions traditionally attributed to enhancer elements. Combining promoter and enhancer functions, the CNS2 element directs its own transcription into two non-coding RNAs. eRNA production is not dependent on the CD69 promoter or CD69 transcription, because sufficient levels of POLR2A are reached at CNS2 element to maintain their production; thus, this element acts as an independent structural and functional transcriptional unit.

We found a positive regulatory function of CNS2 through analyzing its role in the human genomic context. However, in studying the CD69 mouse locus, we previously found that CNS2, despite displaying enhancer properties in luciferase reporter assays, inhibit transcription of the hCD2 reporter when it is placed immediately upstream of the promoter in a transgenic construct (Vazquez et al., 2009). In this case, CNS2 function was evaluated in a non-endogenous context with an artificial system that modifies the natural distances between CNS elements and potential interactions with distal regulators, thereby shifting the regulatory landscape. CNS2 has a crucial function in the regulation of CD69 expression, because it is a requirement for the activation of its transcription. However, it is not the only element within the CD69 locus with potential regulatory properties. DNase hypersensitivity and regulatory epigenetic marks have also been detected for CNS3, CNS4 and a non-conserved DNase hypersensitivity region located in intron 1 (Vazquez et al., 2009, 2012), which also have detectable bidirectional promoter activity.

We have studied the endogenous regulatory function of CNS2 in T and B human cell lines since all the studies up to date evaluating the regulatory role of the different conserved non-coding elements contained within CD69 locus have been carried out in the mouse genomic context and analyzed on these cellular lineages. However, we would expect the function of this element to be the same for those hematopoietic lineages where CD69 expression occurs in an inducible manner, such as NK cells, monocytes and macrophages; but also for tissue resident immune cells that show constitutive expression, as we here demonstrate that CNS2 is not only necessary to activate gene expression upon induction but also to sustain basal activity. Human primary T cells do not stand cloning procedures upon nucleofection therefore future studies must rely on different research approaches as the generation of a CNS2 deficient mouse model.

Although promoters are highly similar to enhancer elements in terms of architecture and functionality (Andersson and Sandelin, 2020), they display directionality in defining TSS and transcriptional progression. Enhancer removal often substantially decreases target gene expression, and low levels are retained, owing to basal activity driven by the promoter. However, CNS2 deletion completely abolished CD69 expression, because the levels of protein or mRNA in CNS2-/- mutants were identical to those found when the promoter was deleted.

Thus, neither the CD69 promoter nor the CNS2 element alone can direct gene expression; these results suggest that both regulatory elements stablished a cooperative activity in promoting transcription of the CD69 gene. The promoter function of the CNS2 element is also reinforced by its ability to act alone in directing reporter gene expression in both directions, in contrast to the promoter. This finding is in agreement with published data in which the promoter activity of enhancer elements in reporter assays has been found to be associated with their ability to direct their own transcription into non-coding RNA species bidirectionally in their endogenous genomic context (Mikhaylichenko et al., 2018). In fact, the CNS2 element overlaps with bidirectional CAGE tags, therefore, as previously described, the ability to act as a promoter correlates with CAGE tag frequency (Andersson et al., 2014a).

The CNS2 element has orientation independent properties, as described for enhancers (Fromm and Berg, 1983; Kong et al., 1997), although its ability to function in some orientations is reduced. Several studies have revealed that enhancer capacity is affected by inversion, owing to an altered chromosomal context. Inversion can disrupt cohesion-mediated chromatin domains, thereby altering the genome topology and, consequently, decreasing enhancer or promoter function (Guo et al., 2015; Tsujimura et al., 2015; Spielmann et al., 2018; Laugsch et al., 2019). Although the effect on chromatin architecture can explain the altered activity of CNS2 inversion in the CRISPR-Cas9 edited clones, it is unlikely due to the reduced size of the deletion and the fact that it cannot explain the observed activity of inverted CNS2 in transient transfection assays. One possible explanation may be the presence of a region with unidirectional properties within the CNS2 element, as described for conventional promoters.

Firstly described in 2010 for the β-globin locus, enhancer transcription into non-coding RNA transcripts, termed enhancer derived RNAs (eRNAs), has rapidly become a widely accepted feature of all active enhancers (Andersson et al., 2014a), with a higher validation rate than conventional criteria, as histone modifications or DHSs (Lu et al., 2015). The CNS2 element is transcribed into two different eRNAs whose expression precedes CD69 promoter activation, thus resembling the kinetics described for this type of RNA molecules in different cell activation systems (Lewis et al., 2019). CNS2 eRNAs are not processed or polyadenylated, although a minority of eRNAs have been described to be processed and to have termination sites (Koch et al., 2011; Andersson et al., 2014b; Tsai et al., 2018). Circular RNA showed defined termination sites for sense-RNA, thereby supporting the notion that every eRNA displays two alternative 3′ ends arising from the same TSS (Carninci et al., 2005; Kawaji et al., 2009). The transcription kinetics of CNS2 peaked after 30 min of PMA/IO exposure, displaying a longer half-life than tipically described eRNAs. The antisense CNS2 transcriptions expands for at least 3,5 kb and consequently is longer than reported lengths of non-coding transcripts of this types. Certain eRNAs, generally those that are longer and more stable, have been shown to be involved in complex gene regulatory networks. For example, MUNC, a 2 kb non-coding RNA transcript transcribed from an enhancer located 5 kb from MyoD gene on mouse chromosome 7, regulates Myogenin, located on chromosome 1, in trans (Tsai et al., 2018). In another example, KLK3e, arising from androgen response element III, regulates different Kallikrein genes at the same locus in cis (Hsieh et al., 2014). However, whether these molecules should be classified as eRNAs or as long non-coding RNAs is unclear (Andersson et al., 2014b).

CNS2 transcription occurs bidirectionally from two independent TSS at similar levels, with no gene direction bias. We demonstrated that the induction of eRNAs precedes CD69 mRNA expression, and their production is not dependent on the presence of the promoter region or its activity. Thus, as with the α-globin locus (Vernimmen, 2014), the ability of CNS2 to induce self-transcription appears to be independent of the enhancer sequence itself, whereas in other cases, eRNA production appears to be dependent on the promoter (Kim et al., 2010). Nevertheless, the decrease in POLR2A binding to the promoter in the absence of CNS2 suggests an interaction between both regulatory regions, thereby probably confirming the observed synergistic cooperation between elements in inducing CD69 transcription.

## Data Availability Statement

The original contributions presented in the study are included in the article/Supplementary Material, further inquiries can be directed to the corresponding author.

## Author Contributions

PL and JR-A designed the study. JR-A performed the experiments. MF, EL, and LN provided assistance with experimental design and data analysis. SR-P and RT-R helped with CRISPR-Cas9 experiments. JR-A and PL wrote the manuscript. All authors contributed to the article and approved the submitted version.

## Conflict of Interest

The authors declare that the research was conducted in the absence of any commercial or financial relationships that could be construed as a potential conflict of interest.
