# Supplementary material for: Functional Characterization of a Dual Enhancer/Promoter Regulatory Element Leading Human CD69 Expression
Source: Front Genet. 2020 Oct 27;11:552949. doi: 10.3389/fgene.2020.552949 (PMC7652794; doi:10.3389/fgene.2020.552949)
Supplement: Supplementary file 1 [file Table_1.DOCX]

Supplementary figure 1

A)


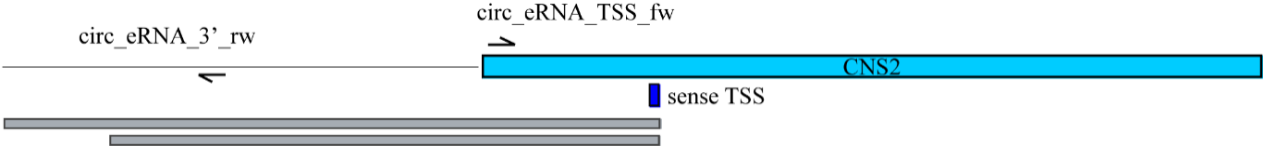


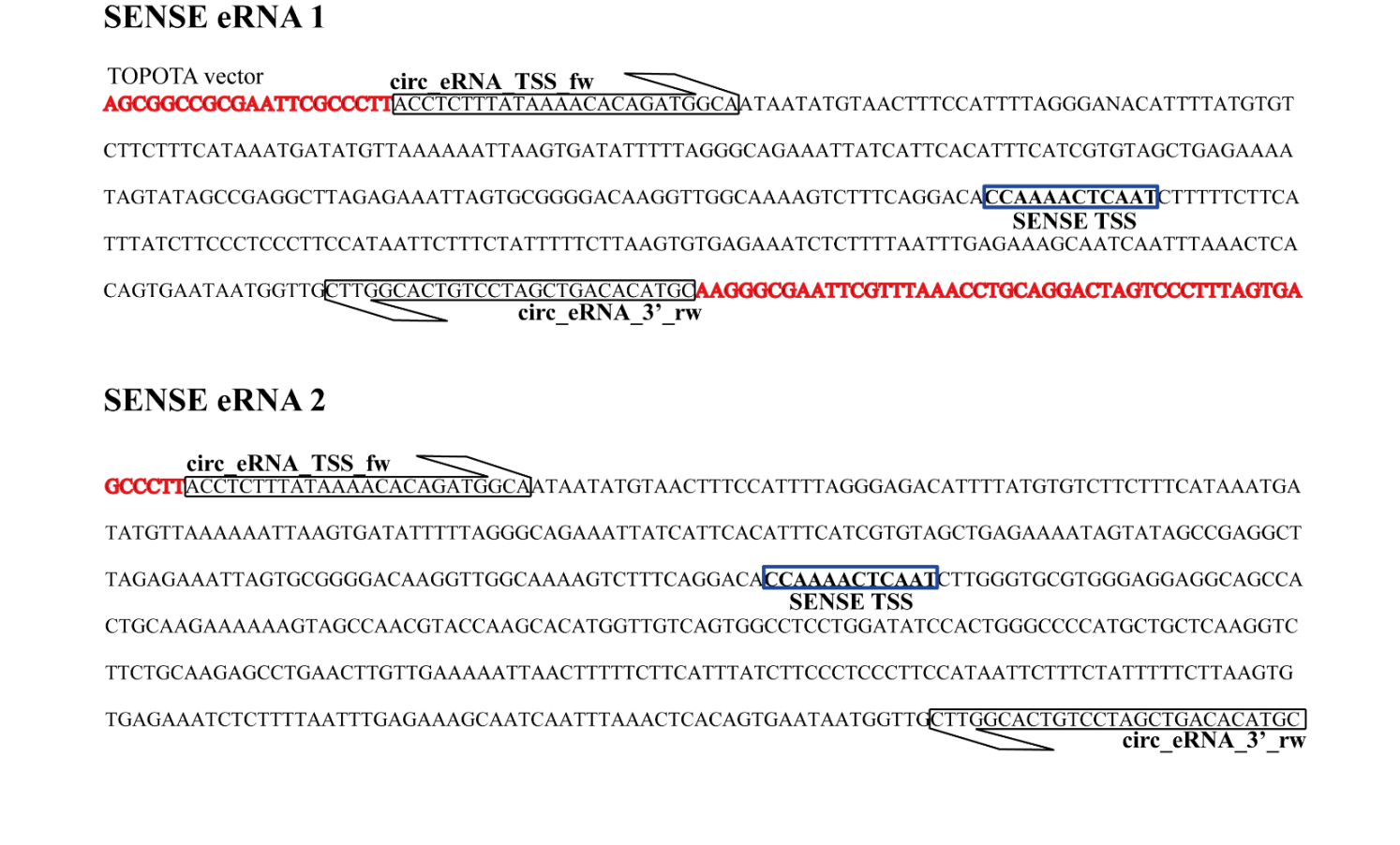


Sense eRNA characterization by the circular RNA technique. Primers position within the CNS2 region are indicated. Sequences of PCR products cloned in TOPOTA vector (red sequence) obtained for each eRNA are shown.

B)


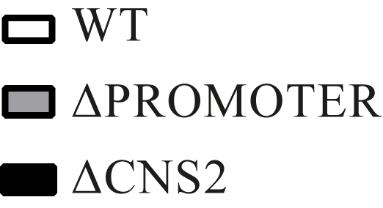

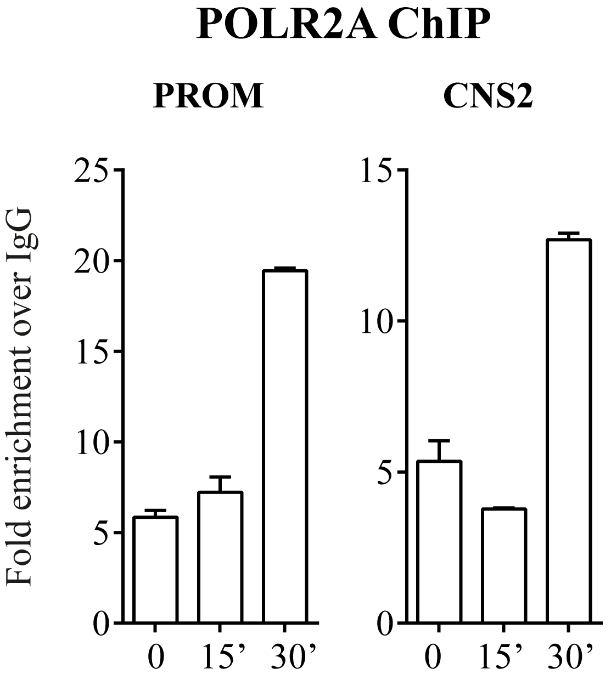


POLR2A binding at CD69 promoter and CNS2 regulatory element assayed by ChIP at the basal state and after 15 and 30 minutes stimulation with PMA/IO. Data represent mean ± SEM of two different technical duplicates.
